# Supplementary material for: Identification of Immunoglobulin Gene Rearrangement Biomarkers in Multiple Myeloma through cfDNA-Based Liquid Biopsy Using tchDNA-Seq
Source: Cancers (Basel). 2023 May 25;15(11):2911. doi: 10.3390/cancers15112911 (PMC10251986; doi:10.3390/cancers15112911)
Supplement: Supplementary file 1 [file cancers-15-02911-s001.zip › Supp.TableS1_perspective.pdf]

Supp. Table S1. Summary of the 43 genes included in the targeted capture-hybridization DNA panel.

# Genes				
ADAMTS7	DDB1	HIST1H1E	PDZK1	TRIM42
ATM	DGKK	IRF4	PGR	XBP1
ATR	DIS3	KRAS	PRKD2	ZFHX4
BCL7A	EGR1	LTB	PRMD1	
BRAF	ERCC1	MAX	PSMC2	
CCND1	FAM46C	MLH1	PSMC6	
CD19	FGFR3	MYO10	PSMD1	
CDKN2A	FGFR4	NFBK2	RB1	
CDKN2C	HIST1H1B	NRAS	TP53	
CYLD	HIST1H1C	NRM	TRAF3	
