# Supplementary material for: Identification of Immunoglobulin Gene Rearrangement Biomarkers in Multiple Myeloma through cfDNA-Based Liquid Biopsy Using tchDNA-Seq
Source: Cancers (Basel). 2023 May 25;15(11):2911. doi: 10.3390/cancers15112911 (PMC10251986; doi:10.3390/cancers15112911)
Supplement: Supplementary file 1 [file cancers-15-02911-s001.zip › Supp.TableS2_perspective.pdf]

Supp. Table S2. Summary of the frequency and alteration data collected on all sample fractions analyzed.

Patient ID	Components	Alteration	Mutation	VAF PPC	VAF cfDNA	VAF BM
MM61	PPC+BM	KRAS	p.Gly12Asp	0.037	-	0.01
	PPC+BM	NRAS	p.Gln61Arg	0.015	-	0.023
	PPC+BM	NRAS	p.Gln61Lys	0.023	-	0.012
	PPC+BM	NRAS	p.Gly12Cys	0.025	-	0.004
	PPC+BM	IGHV4_D6_J4	-	0.065	-	0.211
	PPC+BM	IGKV1_J4	-	0.229	-	0.246
	PPC+BM	IGKV1_J5	-	0.667	-	0.439
MM63	PPC+BM	KRAS	p.Gln61His	0.199	-	0.005
	PPC+BM	IGHV2_D2_J5	-	0.429	-	0
	PPC+BM	IGHV3_Unk_J4	-	0.019	-	0
	PPC+BM	IGKV1_J4	-	0.014	-	0
	PPC+BM	IGKV1_J4	-	0.514	-	0
MM67	PPC+BM	t(11,14)	-	0.014	-	0.026
	PPC+BM	ZFHX4	p.Gly672Ser	0.542	-	0.555
MM88	PPC+BM	BRAF	p.Lys601Asn	0.013	-	0.004
	PPC+BM	BRAF	p.Phe595Leu	0.047	-	0.016
	PPC+BM	NRAS	p.Gln61His	0.283	-	0.033
	PPC+BM	PGR	p.Leu495Arg	0.024	-	-
	PPC+BM	IGHV1_D1_J4	-	0	-	0.11
	PPC+BM	IGHV3_D4_J6	-	0.993	-	0.644
	PPC+BM	IGH_D5_J4	-	0	-	0.096
MM99	PPC+BM	NRAS	p.Gly13Asp	0.067	-	0.03
	PPC+BM	TENT5C	p.Arg301GlufsTer25	0.069	-	0.006
	PPC+BM	TENT5C	p.Ile186GlufsTer12	0.103	-	0.038
	PPC+BM	TRAF3	p.Cys76Phe	0.159	-	0.048
	PPC+BM	TRAF3	p.Ser84Ter	0.078	-	0.032
	PPC+BM	IGHV3_D3_J4	-	0.954	-	0.759
	PPC+BM	IGHV3_D4_J4	-	0.011	-	0
	PPC+BM	IGKV1_J1	-	0.011	-	0
MM101	PPC+BM	CYLD	p.Cys799Ser	0.051	-	0
	PPC+BM	CYLD	p.Thr35HisfsTer12	0.108	-	0.02
	PPC+BM	t(11,14)	-	0.804	-	0.309
	PPC+BM	IGHV2_D4_J3	-	0	-	0.024
	PPC+BM	IGHV3_D4_J5	-	0.985	-	0.919
	PPC+BM	IGKV1_J3	-	0	-	0.024
MM104	PPC+BM	CCND1	p.Ser41Pro	0.059	-	0.179
	PPC+BM	DIS3	p.Asp479Gly	0.044	-	0.125
	PPC+BM	IRF4	p.Lys123Arg	0.052	-	0.144
	PPC+BM	KRAS	p.Gln61His	0.03	-	0.11
	PPC+BM	t(11,14)	-	0.04	-	0.161
	PPC+BM	IGHV1_Unk_J5	-	0	-	0.023
	PPC+BM	IGHV3_D4_J4	-	0.571	-	0.764
	PPC+BM	IGHV3_D6_J4	-	0.104	-	0
	PPC+BM	IGKV1_J4	-	0.221	-	0.155
MM108	PPC+BM	BRAF	p.Val600Glu	0.022	-	0.017
	PPC+BM	IGHV1_D4_J4	-	0.8	-	0.233
	PPC+BM	IGHV1_Unk_J5	-	0	-	0.116
	PPC+BM	IGKV1_J2	-	0	-	0.116

Supp. Table S2. *Cont.*

Patient ID	Components	Alteration	Mutation	VAF PPC	VAF cfDNA	VAF BM
MM111	PPC+BM	NRAS	p.Gly13Arg	0.136	-	0.02
	PPC+BM	IGHV3_D1_J6	-	0.019	-	0
	PPC+BM	IGHV4_D2_J6	-	0	-	0.125
	PPC+BM	IGHV4_D5_J4	-	0.647	-	0.375
	PPC+BM	IGKV1_J2	-	0.027	-	0
	PPC+BM	IGKV1_J2	-	0.252	-	0
MM119	PPC+BM	IRF4	p.Asn102Lys	0.318	-	0.142
	PPC+BM	IGHV3_D3_J3	-	1	-	0.712
	PPC+BM	IGKV1_J4	-	0	-	0.288
MM126	PPC+BM	NRAS	p.Gln61Leu	0.13	-	0.04
	PPC+BM	t(11,14)	-	0.147	-	0.05
	PPC+BM	IGHV1_D1_J4	-	0.888	-	0.895
MM103	PPC+BM+cfDNA	NRAS	p.Gln61Arg	0.093	0.001	0.019
MM107	PPC+BM+cfDNA	KRAS	p.Gln61His	0.232	0,04	0.029
	PPC+BM+cfDNA	IGHV3_D1_J6	-	0.075	0	0
	PPC+BM+cfDNA	IGHV3_D6_J4	-	0	0	0.125
	PPC+BM+cfDNA	IGHV3_Unk_J6	-	0.209	0	0
	PPC+BM+cfDNA	IGHV4_D2_J4	-	0	0	0.2
	PPC+BM+cfDNA	IGKV1_J3	-	0.552	0	0.15
MM117	PPC+BM+cfDNA	BRAF	p.Arg735Leu	0.017	0	0.002
	PPC+BM+cfDNA	BRAF	p.Gly455Trp	0.014	0.003	0
	PPC+BM+cfDNA	DIS3	p.Asp479Glu	0.059	0.001	0.012
	PPC+BM+cfDNA	KRAS	p.Gln61His	0.145	0	0.022
	PPC+BM+cfDNA	KRAS	p.Gly12Val	0.065	0	0.01
	PPC+BM+cfDNA	RB1	p.Pro371Thr	0.013	0.005	0
	PPC+BM+cfDNA	IGHV3_D2_J2	-	0.008	0	0
	PPC+BM+cfDNA	IGHV3_D2_J2	-	0.06	0	0
	PPC+BM+cfDNA	IGHV3_Unk_J6	-	0.76	0	0.781
	PPC+BM+cfDNA	IGKV1_J5	-	0.15	0	0.098
	PPC+BM+cfDNA	t(11,14)	-	0.527	0	0.076
MM125	PPC+BM+cfDNA	NRAS	p.Gln61Arg	0.184	0.006	0.267
	PPC+BM+cfDNA	TENT5C	p.Leu29His	0.039	0.001	0.257
	PPC+BM+cfDNA	IGHV3_D2_J4	-	0.123	0	0.113
	PPC+BM+cfDNA	IGKV1_J4	-	0.857	1	0.864
MM129	PPC+BM+cfDNA	PRKD2	p.Ala578Ser	0.015	0.001	0.004
	PPC+BM+cfDNA	IGHV1/3_D2_J6	-	0.02	0	0
	PPC+BM+cfDNA	IGHV3_D4_J4	-	0.815	0.889	0.634
	PPC+BM+cfDNA	IGKV3_J4	-	0.156	0	0.254
MM36	cfDNA	BRAF	p.Gly32Arg	-	0.081	-
	PPC+cfDNA	KRAS	p.Gln61His	0.172	0.0005	-
	PPC+cfDNA	KRAS	p.Gly12Asp	0.066	0.001	-
	PPC+cfDNA	IGHV3_Unk_J4	-	0.875	0	-
MM46	PPC+cfDNA	CDKN2C	p.Gly137Val	0.23	0.01	-
	PPC+cfDNA	KRAS	p.Gly12Asp	0.51	0.033	-
	PPC+cfDNA	TP53	p.Arg213Gln	0.12	0.008	-
	PPC+cfDNA	IGHV1_D7_J1	-	0	0.571	-

Supp. Table S2. *Cont.*

Patient ID	Components	Alteration	Mutation	VAF PPC	VAF cfDNA	VAF BM
MM57	PPC+cfDNA	BRAF	p.Glu228Ter	0.011	0.0003	-
	PPC+cfDNA	RB1	p.Ser816Ter	0.043	0.002	-
	PPC+cfDNA	IGHV3_D4_J4	-	0.529	0	-
	PPC+cfDNA	IGHV4_D5/2_J5/4	-	0.451	0	-
MM60	PPC+cfDNA	KRAS	p.Gly12Asp	0.093	0.0009	-
	PPC+cfDNA	IGHV3_D6_J4	-	0	0.281	-
	PPC+cfDNA	IGHV4_D3_J4/5	-	0	0.188	-
	PPC+cfDNA	IGHV4_D3_J4/5	-	0.824	0	-
MM65	PPC+cfDNA	BRAF	p.Val600Glu	0.378	0.006	-
	PPC+cfDNA	IGHV3_D6_J4	-	0	0.185	-
	PPC+cfDNA	IGHV4_D3_J4	-	0	0.185	-
	PPC+cfDNA	IGHV5_D2_J6	-	0.738	0	-
	PPC+cfDNA	IGKV1_J4	-	0.119	0.185	-
MM100	PPC+cfDNA	DIS3	p.Cys814Trp	0.57	0.04	-
	PPC+cfDNA	NRAS	p.Gln61Arg	0.288	0.034	-
	PPC+cfDNA	t(11,14)	-	0.085	0.003	-
	PPC+cfDNA	IGHV4_D1_J4	-	0.919	0.769	-
	PPC+cfDNA	IGHV1_D7_J1	-	0	0.154	-
	PPC	IGKV1_J2	-	0.044	-	-
MM118	PPC+cfDNA	BRAF	p.Val600Glu	0.104	0.001	-
	PPC+cfDNA	IGHV3_D1_J4	-	0.368	0	-

Abbreviations: PPC—pathogenic plasma cells; BM—bone marrow; cfDNA—cell-free DNA; VAF—variant allele frequency.
